# Notes and News

**Published:** 1904-10-08

**Authors:** 


### NOTES AND NEWS.

Two villas for male and female patients have been added to the Murray's Royal Asylum, Perth, and also a new chapel. The villas cost about £2,200 each.

A NEW isolation hospital has been opened for the Hereford and Westley Rural District Councils. The buildings are arranged in four blocks. The wards each accommodate four patients, and there is an observation ward. The site cost £175, the building £2,050, water supply £240, architect £128, furnishing £140, and fencing £30. It is interesting when such details of expenditure are forthcoming.

AT the opening ceremony of the Medical Session at King's College Hospital Dr. Buzzard made several announcements as to the future connection of the Medical School with the New Hospital when established at Camberwell. Except in name there would probably be a complete severance between the College and the Hospital, and most certainly as far as preliminary subjects were concerned, but accommodation could be provided at the hospital for the teaching of medicine, surgery, obstetric medicine and gynæcology, pathology, forensic medicine, hygiene and the more special clinical subjects, the administration of anæsthetics, orthopædics, *x*-ray and cognate work. He also stated that the student who commenced his studies at King's College would not be unduly tempted to continue them at Camberwell.

HOSPITAL SATURDAY is to be held on Saturday, October 15th. Last year the collection was a record one, amounting to £23,674, which exceeded by £710 that of any previous year. The action of the fund in abolishing street collections has been thus fully justified, its popularity has been increased, and we hope that this year's record will be thoroughly satisfactory. The Hospital Saturday Fund is pre-eminently the working man's fund, as its main support is derived from the workshops. It has its own Surgical Appliance and Ambulance Department and Convalescent Home. It is largely worked by local committees, and the more energetic these committees are the more likely is the Fund to extend its sphere of interest—a development which could never have been hoped for in the old days of the casual and soon-forgotten gift secured by street collectors.

A CONSUMPTIVE hospital has been opened at Blencathra for the Cumberland branch of the National Association for the Prevention of Consumption. The establishment contains 20 beds, and the cost has been £8,500. Sir William Broadbent delivered an address at the opening ceremony.

THE Odontological Society of Great Britain is prepared to receive applications for grants in aid of the furtherance of scientific research in connection with dentistry. Particulars and forms of application will be supplied by the Honorary Secretary, Scientific Research Committee, 20 Hanover Square, W.

THE Exhibition of the Institution of Hygiene opened last week by Sir Joseph Fayrer in Devonshire Street is under the auspices of a medical council and is intended to serve as a medium between supply and demand, where inventors and purveyors can exhibit novelties and the medical profession and the public can keep themselves acquainted with all modern improvements and inventions connected with hygiene.

THE chairman of the London Hospital has taken another opportunity to disclaim in the Press the association of that hospital with the "Penny Fund" run by "Public Opinion." Anyone who is attracted by the offer of prizes made by that paper, need not flatter themselves that their action will be mistaken for charity. The "League of Mercy" offers all would-be contributors of small sums to the hospitals a means of giving the whole of their contribution without deduction, so there is no need of a commercial agency, which deducts 25 per cent of all the money it collects. The idea of a hospital stamp originated with King Edward's Hospital Fund years ago.

THE Leeds Corporation have erected two fever hospitals, and last week the larger of the two, at Seacroft, was opened by the Lord Mayor of Leeds. The Seacroft Hospital for 452 patients is arranged partly in pavilions placed in pairs with a short covered way between and partly in single pavilions. These pavilions are on arches. The whole number are connected by these covered ways with the administration buildings which stand central. There is a large water tower and residential houses for those connected with the hospital. The cost of this hospital and that at Killingsbeck together has been about £361,000. The Seacroft Hospital stands on a site of forty acres. Close at hand is the Killingsbeck Hospital for small-pox, which has accommodation for 90 cases in the wards, and 14 isolation cases.

IT is reported that New York is about to issue very stringent regulations as to what is to be regarded as adulteration of food and drinks. In brief, nothing is to profess to be what it is not, and nothing must be added to an article which shall give it an appearance of better quality than it actually possesses. This, of course, would strike a blow at all colouring, however innocuous, and whiskey manufacturers especially are said to feel considerable alarm. If the consumer could really rely on the description given of the food and drink he buys, it would be a real boon to the present generation. But we suspect that there are not a few purchasers who are as little anxious to investigate into the constituents of cheap provisions, as is the purveyor to advertise them.

